# Biodegradable Thermoplastic Materials with Application in the Manufacture of Bags Without Synthetic Polymers

**DOI:** 10.3390/polym17030356

**Published:** 2025-01-28

**Authors:** Denisa Nicoleta Airinei, Cristina Modrogan, Oanamari Daniela Orbuleț, Annette Madelene Dǎncilǎ, Magdalena Boşomoiu, Cristian Matei

**Affiliations:** 1 Department of Analytical Chemistry and Environmental Engineering, Faculty of Chemical Engineering and Biotechnologies, National University of Science and Technology Politehnica Bucharest, 1-7 Gheorghe Polizu Street, 011061 Bucharest, Romania; denisa.airinei@yahoo.com (D.N.A.); cristina.modrogan@upb.ro (C.M.); oanamari.orbulet@upb.ro (O.D.O.); magdalena.bosomoiu@upb.ro (M.B.); 2 Department of Inorganic Chemistry, Physical Chemistry and Electrochemistry, Faculty of Chemical Engineering and Biotechnology, National University of Science and Technology Politehnica Bucharest, 1-7 Gheorghe Polizu Street, 011061 Bucharest, Romania; cristian.matei@upb.ro

**Keywords:** environmental impact, thermoplastic materials, biodegradable bag, starch, PLA

## Abstract

Non-degradable plastic bags are a major contributor to marine and soil pollution. They represent a significant percentage of the generated solid waste and can last for hundreds of years in the environment. The aim of the present study was to find alternatives to conventional non-degradable plastic bags by obtaining biodegradable and compostable bags starting from simple materials like starch, poly(lactic acid) (PLA), and glycerol. Increasing the strength and hardness of the polymer was achieved by adding a mineral (talcum). The preliminary studies indicated that two compositions are suitable for advanced testing to produce the initial granular material. These materials were tested for the determination of melt flow index (MFI), Fourier Transform Infrared Spectroscopy (FTIR), and the polymers response to heating (thermogravimetric analysis, TGA and differential scanning calorimetry, DSC). The polymer biodegradability was evaluated by burial in two types of soil. The obtained results were compared with the same set of experiments performed on conventional polyethylene bags. After three months in the soil, only the materials synthesized in this study show signs of accentuated degradation while polyethylene bags are still intact. The surface morphology was explored by scanning electron microscopy (SEM). The results indicated that the biodegradable thermoplastic material meets the requirements of the European standard EN13432/2002 regarding compostable and biodegradable packaging.

## 1. Introduction

Non-degradable plastic is responsible for extensive environmental pollution; it is estimated that nearly half of the marine plastic waste is mismanaged plastic bags [1]. During the processing at waste management facilities, non-biodegradable plastic bags mechanically break into smaller parts as macro (>5 mm), micro (<5 mm), and nano (<1 µm) particles, which are resistant to subsequent degradation. The current methods used for waste disposal are landfilling and incineration. Given the small dimensions, these particles end up in the soil and water. To reduce plastic pollution, a tax on bags was imposed which contributed considerably to the decrease in the use of non-biodegradable plastic bags [2]. However, this only solves partially the problem of large quantities of plastic discarded yearly in the environment [3]. Alternative methods have taken into consideration the manufacturing of bags using biodegradable and compostable polymers, which can be eliminated by simple burial in a compostable pit. Under the action of microorganisms and environmental conditions, they biodegrade within several months without generating harmful substances [4,5,6,7].

**Figure 1:**
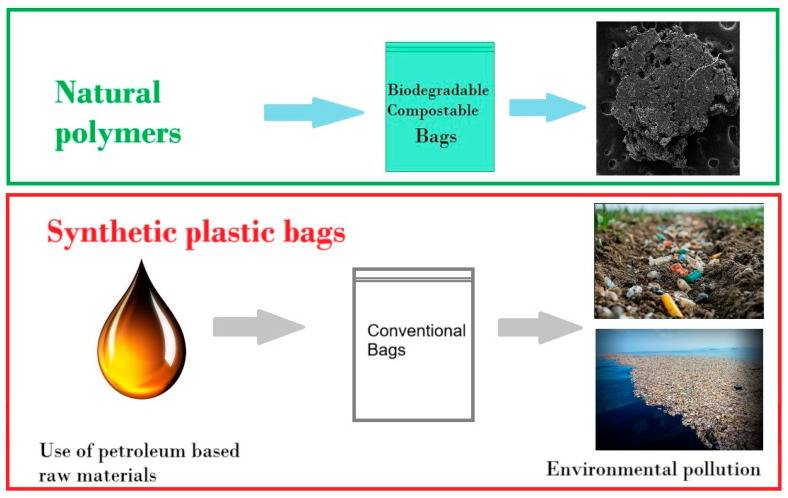
Biodegradable vs. conventional bags and environmental impact.

Another major environmental impact related to conventional plastic bags is the utilization of fossil fuels to synthesize the polymer (Figure 1). Synthetic polymer manufacturing from fossil fuels is responsible for atmospheric, water, and soil pollution. The direct consequence of atmospheric pollution is climate change, while water and soil pollution affect biodiversity [1,8,9]. However, because the synthesis of bioplastics is under development, large quantities of plastic materials are still produced from petroleum [10,11]. Bioplastic materials are classified by their raw source and biodegradability into (i) natural raw materials and biodegradable, (ii) natural raw materials but non-biodegradable, and (iii) synthetic raw materials (e.g., polymers from petroleum) and biodegradable [12].

In the Table 1 are given for comparison purposes, the compositions of several ma-terials tested for the manufacturing of biodegradable plastic bags. The authors who performed the degradation tests on commercial biodegradable bags have underlined that in some cases the biodegradation process is difficult or incomplete [13,14], while in some cases hazardous substances are released [15,16].

Biodegradable plastic bags manufactured from synthetic polymers are susceptible to contain low traces of toxic elements (e.g., Cr, Co, Ba, Zn, Mn, V, Cd, Mo) [21]; these can accumulate and have a negative impact on an ecosystem.

An alternative to remediate the above-mentioned problems is to employ natural polymers. It has been shown that natural polymers (like starch, alginate, etc.) can be used for biodegradable plastic bag manufacturing [17]. Starch can be efficiently recovered from agricultural residues, agricultural by-products, or wastewater generated during food processing [22]. An example is the equipment designed to recuperate the starch contained in the water that is used in the technological flux of potato processing [23]. Starch can be included in the formulation of composites, especially for improving the tensile strength and thermal properties of final materials [24,25].

Poly(lactic acid) (PLA) is currently regarded as an environmentally friendly alternative to produce bioplastic that replaces petroleum-derived plastic. The monomer, lactic acid is synthesized from renewable sources (sugarcane, potato, cashew, apple bagasse, maize, and corn) by fermentation; the enzymatic route is followed to produce PLA [26,27]. PLA has poor mechanical properties, and to increase its mechanical resistance, UV light resistance, and enhance its antibacterial or waterproof properties, it needs to be incorporated into composite materials that can be further used in different applications [28,29,30,31,32]. Adding tapioca starch was found to improve the mechanical properties of the resulting packaging material [33].

Talcum powder has been previously used for its capacity to produce reinforced talcum—wheat starch bio-composite [34,35]. The structure of talcum is composed of octahedral magnesium sites layered between silica atoms and shared common oxygen atoms; moreover, its structure consists of two surfaces one hydrophobic due to Si—O—Si groups, and one hydrophilic due to Si—OH groups [36]. This layered structure makes talcum suitable for incorporation into composite materials.

This study aims to test new thermoplastic materials synthesized from renewable sources, without using fossil fuel-based raw materials, and compare their performance with that of conventional synthetic polymers (high-density polyethylene) in the manufacture of plastic bags. The thermoplastic materials were obtained by applying shear forces at high temperature to a mixture of corn starch, PLA, and talcum in the presence of a plasticizer (e.g., glycerol to increase the material’s flexibility). Talcum was added to improve the mechanical resistance of a thermoplastic film. To the best of our knowledge, to our knowledge, a similar study has not been published previously. Based on its composition, the final thermoplastic material is expected to biodegrade completely to become a nutrient-rich compost and have no impact on the environment (on soil, plants, organisms, and water) compared to similar materials obtained from synthetic and natural polymers [37]. This was verified by conducting experiments by burying thermoplastic materials in the soil.

## 2. Materials and Methods

### 2.1. Materials

The reagents used for the preparation of the biodegradable and compostable thermoplastic materials are provided by Sigma Aldrich (Burlington, MA, USA): corn starch (p.a.), and by Fluka (Morris Town, NJ, USA): PLA (p.a.), glycerol (99.5%), and talcum powder (98%).

### 2.2. Methodology for Bio-Composite Material Synthesis

Corn starch, polylactic acid, and talcum powder were mixed to obtain a good dispersion of the particles. Glycerol and distilled water were added to the mixture of the three powders. Thus, the sample obtained was mixed for 24 h at 180–190 rpm. At the end of 24 h, the resulting mixture was processed at a temperature of 140 °C for 15 min until the excess water evaporated completely. The resulting material was granulated by injection, the size of the granules being between 0.15 and 0.2 mm and was then introduced to the extruder for the formation of the thermoplastic polymer film.

**Figure 2:**
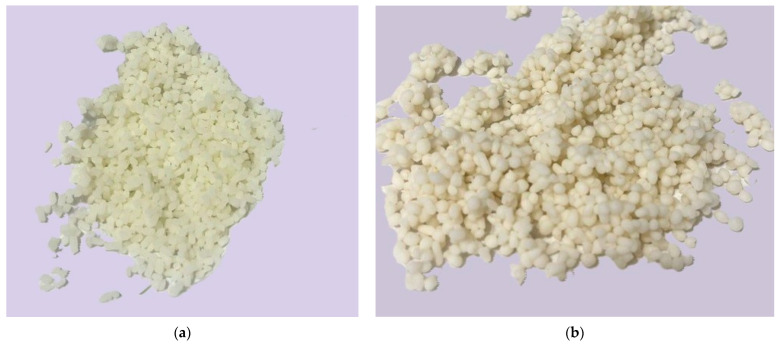
Granular thermoplastic composite: (**a**) sample 1; (**b**) sample 2.

To produce the granular thermoplastic powder, two compositions, given in Table 2, have been tested. The resulting granular composite materials are illustrated in Figure 2. For each composition the tests were performed in triplicate.

These compositions were obtained using the factorial design method, an efficient statistical method for investigating the effect of multiple factors (in this case composition) on the outcome [38,39]. Following preliminary tests, mechanical properties (strength, flexibility), processability during mixing, and granule formation were evaluated. The choice of weights was governed by the functional role of each material:

- Starch (25–30 g) provides the basic structure, but too little (such as 5 g) would make the composition unstable;
- PLA (5–25 g) influences the mechanical properties, and the lower proportion in sample 1 is intended to maintain biodegradability, while sample 2 tests durability at a higher proportion;
- Glycerol (20–30 mL) is used for flexibility; lower amounts can create a stiff material, and higher amounts could compromise the structure.

The reduced talcum weight reflects its auxiliary role in improving the processability and thermal stability of the final material. The higher amount of talcum in sample 1 (3 g) can be justified by the fact that it contains less PLA and requires additional support for stability. In sample 2, the talcum is reduced (2 g) because the composition with more PLA has already improved the thermal and mechanical stability.

### 2.3. Methods Used for the Characterization of the Thermoplastic Composites

The obtained thermoplastic composites were characterized by Fourier Transform Infrared (FTIR) analysis, determination of melt flow index (MFI), polymers response to heating (TGA and DSC), and mechanical tests.

FTIR spectra were recorded using a Bruker Tensor 27 spectrometer (Bruker, Karlsruhe, Germany) equipped with a universal attenuated total reflectance. The spectra were recorded between 4000 and 480 cm^−1^ wave number range.

The melt flow index was measured using a plastometer PCE-MFI 400 (PCE, Meschede, Germany). The flow index represents the amount of polymer melt that flows through a nozzle, at a certain temperature, under the weight of standard mass pressing on a piston; this is a measurement of the polymer melt flow rate and viscosity. This measurement is performed according to ISO 1133 or ASTM 1238. The temperature and reference weights of the test masses are chosen specifically to the polymer tested to best characterize the rheological properties of the analyzed sample. In this study, the tests were carried out at a temperature of 210 °C, and the weight of the reference mass was 2.16 kg.

TGA was carried out using a STA 449 C Jupiter equipment from Netzsch (Gottinger, Germany), with heating range from 25 to 800 °C at a constant heating rate of 10 °C/min and continuous air flow.

For DSC experiments, samples weighing 22.900 mg were prepared; a STA 449 C Jupiter apparatus from Netzsch (Germany) differential scanning calorimeter was used. The temperature scans were performed between 20 and 200 °C with a heating rate of 10 degrees/min, in dry air atmosphere.

The hardness was measured using the digital Shore hardness tester SAUTER HEA 100, Kern (Ballingen, Germany). To calculate or measure the hardness of a biodegradable bag, the following steps were followed: a piece of the biodegradable bag without defects was cut and the Shore durometer was placed on the surface of the tested material; a constant force of 1.24 N was applied to the material; the value displayed on the durometer was read. The above steps were repeated at several points on the material to obtain an average value. Five measurements were made for each bag. The extremes were eliminated, and an arithmetic mean was made with the remaining values.

To calculate the tensile strength, tensile tests are performed according to a standard, such as ISO 527 [40]. The following steps were followed: a rectangular sample was cut with a width of approximately 10 mm and a length of approximately 100 mm; the sample was placed in the testing machine and its ends were attached to clamps; the machine applied a constant force to stretch the sample, while monitoring the applied force and the elongation of the material. The test continued until the material broke. The measurements were made using a universal automatic machine for tensile and compression testing, Matest 1000 kN.

The process of making the sample bags consisted of hot pressing the granulated material, at a temperature of 140–160 °C, using a minilab Brabender extruder (Duisburg, Germany). This process ensures partial melting of the PLA and the obtaining of a uniform and resistant film.

The samples resulting from the biodegradability test were analyzed by SEM (Scanning Electron Microscopy). The microscope used is a Tescan Vega-3 LMH type that allows the investigation at high vacuum (7·10^−2^ Pa) and electron acceleration of 10 kV.

## 3. Results and Discussions

### 3.1. FTIR

**Figure 3:**
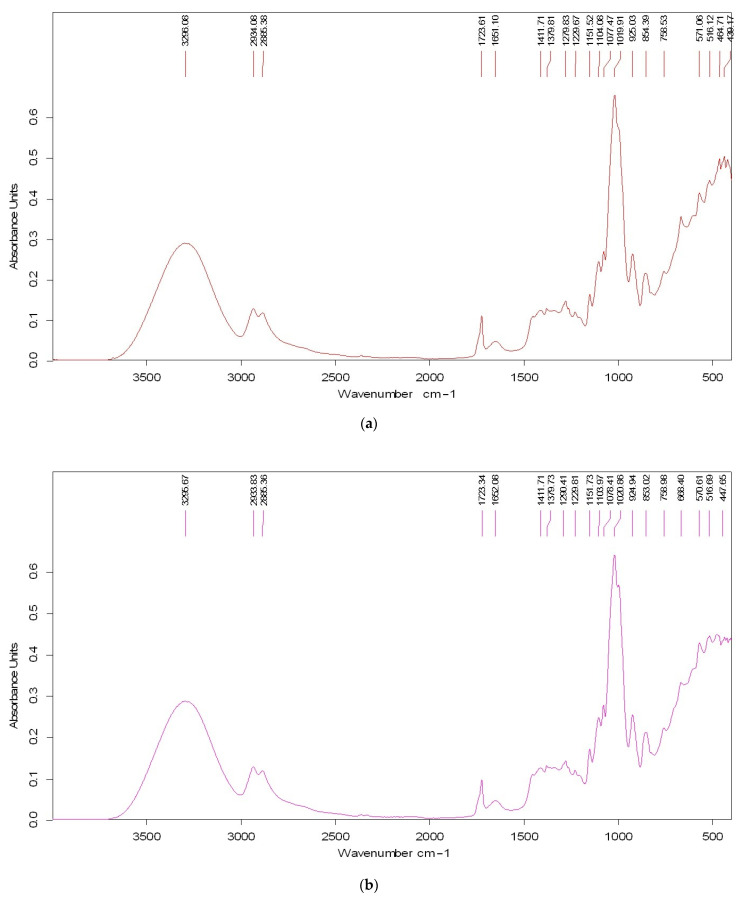
FTIR spectra of thermoplastic materials: (**a**) sample 1; (**b**) sample 2.

FTIR spectra of thermoplastic materials are given in Figure 3. For sample 1 (Figure 3a), the characteristic bands of starch are identified from 3200 to 3600 cm^−1^ (vibration that corresponds to O–H stretching) and from 1000 to 1150 cm^−1^ (attributed to C–O–C and C–O vibrations). PLA characteristic reduced peaks are identified in the interval 1740–1760 cm^−1^ (C=O in esters), while the presence of bands in the 2800–3000 cm^−1^ area is attributed to aliphatic C–H vibrations; the reduced intensity of the peaks is due to the small amount of PLA in the sample [41,42]. The presence of talcum is indicated by the characteristic Si–O vibrations around 960–1050 cm^−1^ [43]. Glycerol has a moderate contribution in the 3200–3400 cm^−1^ region (O–H stretching) and signals assigned to the C–O bond around 1030 cm^−1^. The spectrum of sample 2 reflects also the predominant contributions of starch and glycerol in this composition (Figure 3b). Talcum contributes modestly, highlighted in the region below 600 cm^−1^. The characteristic C-O and Si-O bands (960–1150 cm^−1^ area) are influenced by the presence of starch (the intense signal at 1000 cm^−1^ has been associated with bending vibrations of C–O–H glycosidic bonds) [44].

### 3.2. Melt Flow Index

Melt flow measurements are important to the polymer industry for several reasons. The first is that it gives indications if a thermoplastic material can be extruded, moulded, or rendered in the form of objects of use, through a common process of plastic conversion. In all these processes, the plastic is first melted and then forced to flow through an extrusion die or mould that will give it its final shape. If the flow of the material is not suitable (for example, the polymer does not completely fill the mould or distortions of the extrudate or uneven parts of the profile are produced, or parts are formed that succumb to normal pressures or light impact) it means that the respective material is not appropriate for the possible use assigned. Secondly, an increase in the melt flow from the specified one may indicate a degradation of the polymer molecules, and a decrease may represent the result of the reaction between the molecules or their chaining. Composition errors can cause changes in flow direction. All these molecular changes can cause a loss of impact resistance or chemical resistance, sufficient to affect the final properties of the final object.

**Figure 4:**
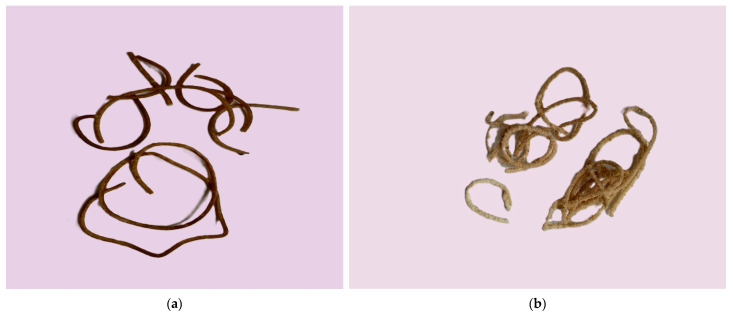
Extruded thermoplastic composite: (**a**) sample 1; (**b**) sample 2.

The resulting thermoplastic extrudates are illustrated in Figure 4.

The values of MFI were calculated for each sample using the formula:

MFI(T, m_nom_) = (600∙m)/t (1)

where T is the temperature of the polymer melt, [°C];

m_nom_—test mass, [kg];
m—average mass of the samples taken, [g];
t—sampling time, [s].

The melt flow rate of a polymer is an indirect measure of molecular weight and viscosity. A low molecular weight results in a high melt flow rate, and an increase in temperature or test mass results in an increase in melt flow due to a decrease in viscosity. The calculated values of MFI are given in Table 3. MFI has a much more pronounced increase for larger test masses, which can be translated into the fact that during injection, the injection pressure must first be increased and then, if the requirements of subsequent use are not met, the injection temperature can also be increased.

The values indicated in Table 3 are similar or superior to other values reported for starch based thermoplastic materials [45]. Among the two tested thermoplastic materials, the composite with lower PLA content has higher moulding ability; however, sample 2 material has also a good moulding ability.

### 3.3. Thermogravimetric Analysis

**Figure 5:**
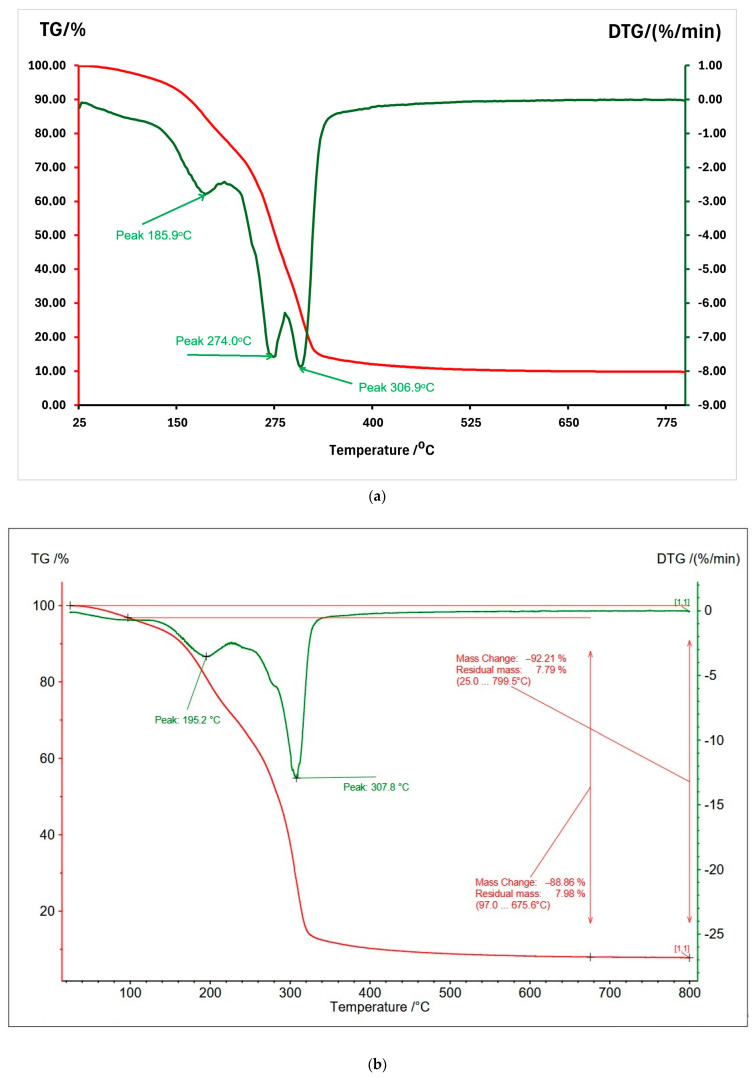
Thermogravimetry analysis (TGA) and derivative thermogravimetric (DTG) curves: (**a**) sample 1; (**b**) sample 2.

The thermal decomposition of sample 1 shows a gradual decrease in mass starting at a temperature of approximately 185.9 °C. This peak is associated with the removal of chemically bound water from the material (Figure 5a). The second major point, at 274.0 °C, corresponds to a more rapid loss of mass, which is attributed to the decomposition of organic polymers (starch, glycerol). The peak at 306.9 °C probably indicates the beginning of the decomposition of the more stable components (PLA) [46]. The final residual mass is likely largely associated with talcum and carbonized materials.

In the temperature range of 25–150 °C, the removal of free and physically bound water (by weak interactions—hydrogen or Van der Waals forces) occurs. In this range, a slight decrease in mass is observed. Chemical dehydration and possible preliminary degradations happen in the temperature range of 150–200 °C. The peaks in the DTG (Derivative Thermogravimetric Curve) at approximately 185.9 °C suggests chemical dehydration (removal of hydroxyl –OH groups, chain scission) of the starch polysaccharides. In the case of glycerol, the molecules may begin to lose water through internal dehydration, leading to the formation of intermediate compounds (such as aldehydes or small acids) [47]. In addition, the starch begins to undergo gelatinization and early thermal degradation [48]. The main mass loss peak is observed at 274 °C (central area on DTG), where the polysaccharide chains in starch undergo glycosidic bond cleavage (cleavage into smaller segments). This process produces volatile compounds, such as monosaccharides (glucose), which further degrade into furfural, hydrocarbons, and carbon dioxide [49]. Glycerol degrades thermally, forming volatile compounds such as acrolein and other organic derivatives [47]. PLA degradation involves an initial depolymerization of its chains. At the temperature range of 300–400 °C, advanced PLA degradation and residue carbonization occur [50]. Above 400 °C, the TGA curve becomes almost horizontal, indicating a stable residual mass composed mainly of carbonaceous residues (generated by starch and PLA) and inert talcum that does not decompose at moderate temperatures.

Sample 2 (Figure 5b), which contains more PLA, shows better thermal stability, with decomposition starting at higher temperatures (~195.2 °C) [50]. The main mass loss occurs between 200 and 350 °C, with a peak temperature of 307.8 °C. Starch decomposition starts around 280–300 °C and is characterized by the breaking of glycosidic bonds and transformation into carbonaceous residues. PLA has a main thermal decomposition around 300 °C, which is confirmed by the DTG peak observed at 307.8 °C [50]. The interaction between residual glycerol and starch/PLA, may contribute also to mass loss at this stage.

Both samples show a stable residual mass of almost 8% attributed to the talcum and carbonaceous residue. The higher starch content of sample 1 contributes to the decrease in overall thermal stability, having earlier decomposition peaks (up to 274 °C). The higher PLA content in sample 2 gives higher thermal stability (~195 °C), but thermal degradation processes are more complex and focus around 307 °C. Sample 2, with more glycerol, shows greater stability at low temperatures and loses water more slowly. This is an additional protection against premature decomposition.

### 3.4. Differential Scanning Calorimetry

Differential scanning calorimetry is a thermal analysis method that focuses on how the heat capacity of a material changes with temperature. Starch containing samples can undergo a series of complex processes during heating (e.g., water loss, melting, gelatinization, change in crystal structure, volume expansion, and molecular degradation) [51].

**Figure 6:**
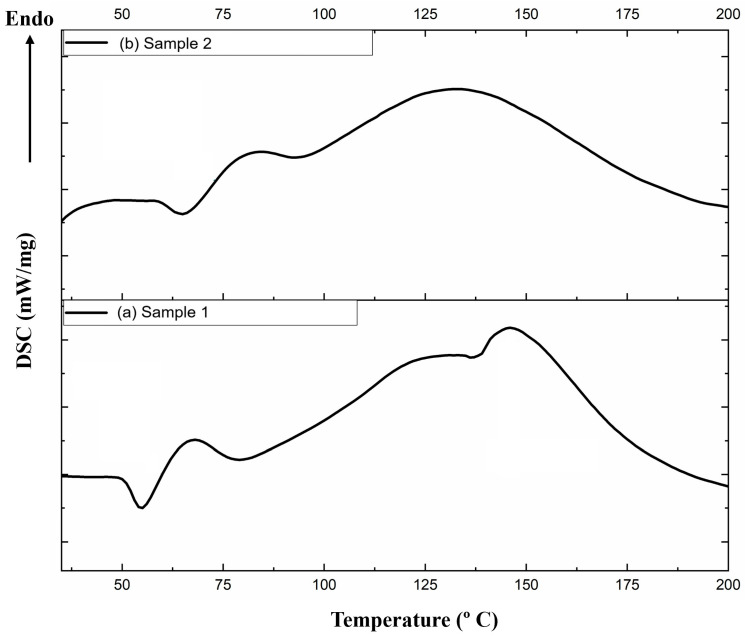
DSC profiles: (**a**) sample 1; (**b**) sample 2.

Sample 1 has an exothermic point at a temperature range between 51 and 52 °C (Figure 6a) while sample 2 at a temperature between 64 and 66 °C (Figure 6b). This corresponds to the loss of water [52] but at a higher temperature compared to sample 1. The temperature difference suggests a more compact structure for sample 2 given by the higher PLA content, which retains water up to higher temperatures. The following points defined for the thermal transitions of the samples are named T_o_ (onset), T_p_ (peak), and T_m_ (melting). Sample 1 has an endothermic peak at a temperature range between T_0_ = 51 °C and T_m_ = 75 °C, with T_p_ = 67 °C (Figure 6a) while sample 2 at a temperature between T_0_ = 64 °C and T_m_ = 88 °C, with T_p_ = 79 °C (Figure 6b), which corresponds to the loss of water. The water loss peak and melting peak are shifted toward higher temperatures for the sample with a higher content of PLA.

With the passage above the Tm point, the macromolecules acquire more mobility. When the polymer reaches a certain temperature, the macromolecules have enough energy to settle into an ordered arrangement forming crystalline zones (crystallites, spherulites). With the passage partially in the crystalline phase, the polymer will re-lease heat (crystallization being an exothermic process). Under these conditions, the heating system of the polymer crucible will provide less heat than before, the heat released by crystallization being also used for the constant increase in the temperature at the imposed speed, which results in the appearance of a little exotherm peak on the graph. Heating the polymer can allow the macromolecules to organize into crystalline areas (lamellas and spherulites), but too much heat can lead to their destruction. Thus, further heating of the polymer above the T_c_ = 139 °C (sample 1), temperature leads to the melting of the crystalline zones. For the crystalline areas of the polymer to melt, a heat input from the outside is needed. This corresponds to T_p2_ = 147 °C for sample 1 and T_p2_ = 127 °C for sample 2. The shifted T_p2_ point towards a lower value for sample 2 is attributed to a decrease in crystallinity for sample 2 [53].

### 3.5. Mechanical Properties

The tensile strength and hardness of a biodegradable bag are two important properties that influence its performance. These properties depend on the material from which the biodegradable bag is made. Tensile strength refers to the ability of the material to withstand tensile or pulling forces without breaking. For a biodegradable bag, this parameter is important to ensure that the bag can withstand weights and pressures during handling or transportation. The hardness of a material refers to its resistance to deformation or scratching. It is an important property for evaluating the stiffness or flexibility of a biodegradable bag.

The Shore test is one of the most common ways to measure the hardness of plastic and elastic materials, including the bioplastics from which biodegradable bags are made. The Shore test involves the use of a Shore durometer, which contains a conical tip or cylinder that penetrates the material with a constant force. The penetration depth is measured and converted into a hardness value, which is expressed on a scale from 0 to 100. The higher the value, the harder the material.

Calculating the tensile strength of a biodegradable bag involves determining the material’s ability to withstand stretching before breaking. Tensile strength is an important property for materials used in bag manufacturing, as it determines how much weight a bag can support before breaking. The values of hardness and tensile strength are given in Table 4.

The results indicate that sample 2 has slightly better mechanical properties although the mechanical properties of sample 1 are within the expected limits.

### 3.6. Comparative Study of the Biodegradation of Thermoplastic and Polyethylene Bags

The experiments of composites buried in soil are usually employed to evaluate the biodegradability of materials [54].

To perform the comparative study, low-density polyethylene (LDPE) bags were chosen as representative for conventional plastic bags that are largely used [55]. To carry out the biodegradability study, two types of soil were used, which were placed in four pots, and the samples were placed inside them for 3 months. The composition of the two types of soil are as follows: soil type 1—flower soil with perlite and dolomite has nitrogen, phosphorus, potassium, water-soluble salt, and trace elements in its composition (Table 5); the composition of type 2 soil—universal soil, which contains nitrogen, phosphorus and potassium but in different percentages (Table 6). The thermoplastic bags were manufactured from the previously characterized materials. The room temperature was set at 25 °C. Soil acts as a source of microorganisms and moisture, both necessary for the biodegradation of materials [56].

**Figure 7:**
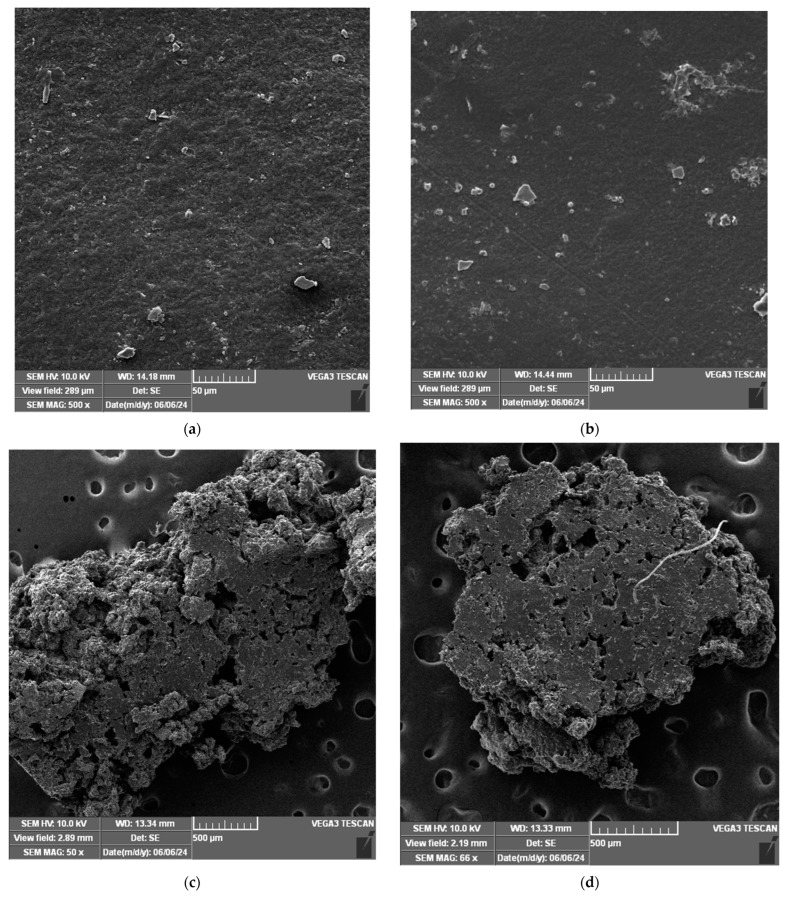
SEM images of bag samples after being buried three months in type 1 soil or type 2 soil types: (**a**) LDPE buried in type 1 soil; (**b**) LDPE buried in type 2 soil; (**c**) thermoplastic sample 2 buried in type 1 soil; (**d**) thermoplastic sample 2 buried in type 2 soil.

After 3 months the bags were dragged out of the soil and analyzed for surface fractures and defects by high-resolution images using SEM analysis. The SEM images are given in Figure 7.

In Figure 7a, the SEM image shows the LDPE surface with a few particles and slight texturing. The small number of deposits and defects suggests limited degradation in type 1 soil. The experiments made using soil type 2 indicate that the primary structure of LDPE remains intact, suggesting high resistance to degradation in this type of soil (Figure 7b). However, the more adhered particles and small agglomerates appear; this is a sign of degradation initiation. These results are attributed to variations in the soil’s characteristics (e.g., composition, moisture, microorganisms, and pH). Experiments conducted with commercial bags made from both natural and fossil fuel-based raw materials have indicated that these bags underwent slow degradation, which is attributed to the presence of petrochemical-derived polymers that are difficult to break down [13,14].

As illustrated in Figure 7a,b, the bag samples made of low-density polyethylene do not show signs of biodegradation, where the surface morphology being specific to a polymer with a ductile nature. A smooth surface is an indication of a high internal plasticization. On the contrary, from Figure 7c,d related to bags made of bioplastic, the presence of areas of accentuated degradation can be observed, the images highlighting a visibly irregular surface.

The image in Figure 7c presents a surface that is highly degraded and rough, with evident porosity. The granular structure and cracks suggest significant degradation, likely caused by biological and chemical factors in the soil. Type 1 soil appears to promote advanced fragmentation of the sample, affecting the integrity of the thermoplastic. Figure 7d shows also a highly degraded surface but appears more compact and less porous. Filament-like material (possibly microorganisms) is visible, indicating more intense biological activity. Type 2 soil appears to affect the sample, but in a more controlled manner, with slightly less advanced degradation compared to type 1 soil.

One way to accelerate the degradation of bags made from synthetic polymers in a mixture with natural polymers is to add chemical compounds (Fe_3_O_4_) [20]. Moreover, it was found that the natural polymers from these bags degrade leaving non-degraded micro and nano particles of synthetic polymers that are a hazard to the environment [15]. Field studies with small-sized film fragments of compostable bags made of natural mixed with synthetic polymers have shown limited degradation of the fragments over a period of 12 months [57].

The findings of these tests indicate that it is preferable to use biodegradable bags made of thermoplastic bio-composites than biodegradable bags made of synthetic polymers (e.g., poly (butylene adipate-co-terephthalate) originated from petrochemicals) [1,18].

According to the European standard EN 13432, the materials tested in this study meet the requirements for packaging materials [19].

## 4. Conclusions

The present experimental study demonstrated that the synthesis of two thermoplastic composites can be successfully used in manufacturing biodegradable and compostable bags that replace the LDPE bags.

DSC analysis indicated differences in thermal behaviour for the two materials; this reflects changes in molecular structure and physical properties of the analysed materials. Sample 1 shows a structure with a higher degree of crystallinity, as evi-denced by its higher melting temperature while sample 2 is characterised by higher temperature interval for the water loss but a lower degree of crystallinity, indicated by the lower Tp2. This was attributed to its higher PLA content.All the substances used for the manufacturing of the biodegradable thermoplastic material meet the requirements of the EN13432/2002 standard [19] regarding packaging that is subjected to biodegradation and composting.

The moulding ability was evaluated by MFI analysis and the results indicated that both materials are suitable for moulding and the suitable parameters were determined. By correlating the obtained results, it was found that the composition of sample 2 is preferable because of its increased mechanical and thermal stability.

The biodegradability study was performed on two samples: one conventional LDPE bag and the other made of thermoplastic material—sample 2, which has the highest mechanical stability. The two materials were subjected to the same degradation conditions, respectively, the same types of soil (1 and 2) for a duration of three months. By comparing the results obtained through SEM analysis, we can see that only the bioplastic material shows significant changes in its structure, indicating fast biodegradability. LDPE shows minimal degradation in both types of soil, being more resistant to environmental conditions. The type 2 soil showed slightly intensified degradation compared to type 1 soil. Compared to LDPE, thermoplastic sample 2 is much more susceptible to degradation, displaying very rough surfaces and porous structures, especially in type 1 soil, where fragmentation is more advanced. The degradation of the thermoplastic material is likely influenced by biological and chemical factors, such as microorganisms and soil composition.

Overall, it can be concluded that thermoplastic material—sample 2 can be a substitute of non-biodegradable/synthetic plastics for obtaining bags. The applications of the starch/PLA thermoplastic materials can be extended beyond the manufacturing of bags given its excellent properties and biodegradability.

## Figures and Tables

**Table 1:** Natural raw materials vs. petrochemical raw materials to produce biodegradable bags.

No.	Composition	Fossil Fuels Based Raw Materials	Observations	References
1.	Starch + PLA + talcum + glycerol	No	Biodegradability tests by composite burial in soil	This work
2.	Sodium alginate Stearic acid	No	Biodegradability confirmed by measuring generated CO_2_	[17]
3.	PLA + PBAT—commercial bag PBAT—poly (butylene adipate-co-terephthalate)	PBAT	According to the tests performed by Ref. [18], the products do not comply with requirements of EN 13432 standard [19]	[18]
4.	PBAT + PLA PBAT + PLA + MD20 PBAT + PLA + St20 MD Magadiite (Na_2_Si_14_O_29_) St—starch	PBAT	Photo degradation tests have showed that the micro and nano particles formed are a hazard for human health and for environment	[15]
5.	Polybutyl terephthalate + starch (EcoPack—Italy) Polybutyl terephthalate + Talcum (Heritage—USA) Polyethylene + CaCO_3_ (MSDS Solutionz Ltd.—UK) Polyethylene + CaCO_3_ + EVA (Relevo—Spain)	Polybutyl terephthalate Polybutyl terephthalate Polyethylene Polyethylene	Compostable bags according to the manufacturer description, biodegradability has not been evaluated by authors	[1]
6.	PLA + polybutylene terephthalate + starch PLA + polybutylene terephthalate + MD	Polybutylene terephthalate Polybutylene terephthalate	Bag additives presented toxicity to microorganisms	[16]
7.	PLA + PBAT + St20	PBAT	Addition of Fenton—like reagents to improve the compost quality of bags—food waste mixtures	[20]
8.	PBAT + PLA	PBAT	Biodegradability tests under anaerobic digestion indicated difficult biodegradability	[13]
9.	PBAT + PLA + starch	PBAT	Biodegradability tests under anaerobic digestion indicated incomplete biodegradability	[14]

**Table 2:** Tested compositions for the granular precursor.

Sample	Corn Starch, g	PLA, g	Talcum, g	Glycerol, mL	Distilled Water, mL
1.	30	5	3	20	40
2.	25	25	2	30	40

**Table 3:** MFI determination.

Sample	m, g	MFI g/10 min.	SD *
1.	0.9230	18.46 ± 0.1878	0.1080
2.	0.6733	13.47 ± 0.0965	0.8655

* SD—standard deviation.

**Table 4:** Mechanical properties.

Sample	Hardness	SD	Tensile Strength, MPa	SD
1	43 ± 1 Shore A	1	29 ± 1	1
2	47 ± 1 Shore A	1	35 ± 2	2

**Table 5:** Composition of type 1 soil.

Elements	Mass Percentage, %
Nitrogen	>0.3
Phosphorus	>0.3
Potassium	>0.3
pH	5–7.5
Water soluble salt	<2.0
Microelements (in natural composition, bound to organic matter)	<0.2

**Table 6:** Composition of type 2 soil.

Elements	Mass Percentage, % *
Nitrogen	1.16
Phosphorus	21 ppm
Potassium	190 ppm
pH	5–7
Wood fibre	0.5–5%
Humus	3.1%
Ash	5%
Moisture	60–70

* If not otherwise specified.

## Data Availability

Data are contained within the article.
